# Diversity in causes and characteristics of drug-induced deaths in an urban setting

**DOI:** 10.1177/1403494812472007

**Published:** 2013-03

**Authors:** Linn Gjersing, Kristine V. Jonassen, Stian Biong, Edle Ravndal, Helge Waal, Jørgen G. Bramness, Thomas Clausen

**Affiliations:** 1 Norwegian Centre for Addiction Research, University of Oslo, Oslo, Norway; 2 Norwegian Institute for Alcohol and Drug Research, Oslo, Norway; 3 Faculty of Health Sciences, Buskerud University College, Buskerud, Norway; 4 Norwegian Institute of Public Health, Oslo, Norway

**Keywords:** Death among drug users, drug use, drug-induced deaths, drug-related deaths, fatal overdose, heroin use, injection drug use, mortality, opiate use, overdose

## Abstract

*Aims*: To assess demographic characteristics, treatment utilization and circumstances of death among those who died from drug-induced deaths in an urban setting and to identify possible subpopulations that should be targeted specifically to further develop preventive public health policies. *Methods*: Subjects (*N* = 231) who died, from drug-induced deaths, in the Norwegian capital Oslo (2006–2008) were identified through the National Cause of Death Registry. Data on toxicology, prison release and contact with health and social services in Oslo were collected. *Results*: Majority of cases were men (78%) and the mean age was 37 years. Nearly all cases (90%) were polydrug intoxications. Heroin was implicated in 67%. Residential address was the most common place of death (67%). Most cases (82%) had been in contact with health and social services in the year before death. Women were 4 years older, more often Oslo residents (82% vs. 64%) and fewer died from heroin intoxication. Non-Oslo residents were younger and more likely to have been found outdoors with heroin as the main intoxicant. Other identified subpopulations were those who died after prison release and those discharged from drug treatment. ***Conclusions*: The findings suggest that the majority of cases could have been available for preventive measures through their contacts with health and social services. Yet, the heterogeneity among cases indicates that such measures need to be multifaceted. Finally, it is important for policymakers and health and social workers in various countries to consider subpopulations such as women and non-city residents when developing public health interventions to prevent overdose deaths**.

## Background

Injecting drug users (IDUs) experience between 10 and 20 times higher mortality compared to the general population [1]. One of the main causes of death is overdose [2]. Commonly it is men in their early to mid-30s who have injected substances over many years who die from an overdose of injected heroin, often in combination with other substances such as alcohol or benzodiazepines [3,4]. Those who die from an overdose have often experienced previous non-fatal overdoses and are also likely to be out of treatment at the time of death [5–7]. The majority of these deaths occur at a residential address [8].

Certain factors, in combination with IV polydrug use, increase vulnerability for drug-induced deaths. More frequent heroin use and heroin purity are two such factors, although purity is only found to have a moderate relationship to overdose [9,10]. Homelessness or shelter use are other such factors [11,12]. Also, recent prison release and discharge from drug-free in-patient treatment are particularly vulnerable times, since people are typically in a state of low tolerance to opioids [13,14]. Overall, there is a whole range of factors that alone or in combination increase the vulnerability for drug-induced deaths and the challenge is to target these factors with preventive measures.

The continuous high annual mortality rate among IDUs in many Western countries suggests that it is difficult to prevent these deaths [15]. Consequently they represent a major public health concern. It is therefore important to assess how to better prevent these deaths and to investigate if and what factors associated with drug-induced deaths differ across settings and time. Thus, in this study, we aimed to examine demographic characteristics, treatment utilization and circumstances of death among those who died from drug-induced deaths in an urban setting (the Norwegian capital Oslo), and to identify any possible subpopulations that should be targeted specifically to further develop preventive public health policies.

## Methods

### Setting

This study was conducted in the Norwegian capital, Oslo. Norway has the highest overdose mortality rate in Scandinavia with 8 overdose deaths per 100,000 inhabitants [16]. The population in Norway is approximately 5 million inhabitants according to Statistics Norway 2012 [17]. One-third of the drug-induced deaths occur in Oslo, which has around 500,000 inhabitants [17,18]. This means there is a disproportionately high rate of overdose deaths in the capital compared to other regions.

The number of IDUs has been estimated to be between 8,800 and 12,500, and heroin is the most commonly injected drug [18]. In response to an increasing number of drug-induced deaths in Norway, opiate maintenance treatment (OMT) was established as a national programme in 1998, and at the end of 2011 there were 6,015 OMT patients [19]. Although the number of drug-induced deaths has been reduced since the introduction of OMT, the annual number remains between 200 and 300 [18].

### Design

This is a retrospective registry study that includes all those between the ages of 15 and 65 that died in Oslo from drug-induced deaths between 1 January 2006 to 31 December 2008 (*n* = 231). In 2006, the number of deaths was 88, in 2007 it was 66 and in 2008 it was 77. These deaths represent 1/3 of all registered drug-induced deaths in Norway during the study period (*n* = 774).

### Inclusion

Subjects were included according to the classification of the European Monitoring Centre for Drugs and Drug Addiction for drug-induced deaths [20]. These are deaths ‘directly due to and generally occur shortly after the consumption of illegal substances, often in combination with other substances such as alcohol or psychoactive medicines’ [20].

Subjects were initially identified through the National Cause of Death Registry, which is a general mortality register. The information was coded as ‘underlying cause of death’. Underlying cause of death is defined as ‘the illness or injury which initiated the train of morbid events leading directly to death or the circumstances of the accident or violence which produced the fatal injury’ [21]. For matching purposes, the data received from the National Cause of Death Registry included full name, personal identification number, date of birth, date of death, postal code for region of death, residential postal code and whether the person had a post-mortem examination (hospital or forensic).

Vital data were cross-linked with other registries and patient/client records. The information was collected from each institution separately either via cross-linked records or searched manually.

Data on toxicology were received from the Institute of Forensic Medicine at the University of Oslo. These data included the place of death and the postal code for the place of death. Place of death included the following variables: ‘residential address’, which included shelters providing long-term accommodation; ‘outdoors’, which included parking houses and public toilets; and ‘institutions’, which included hospitals, drug treatment facilities and prisons. Although only one substance was considered to be the main intoxicant by the pathologist, a person could have several other substances in their blood that may have contributed to the death. Information on both main intoxicant and other substances were collected.

The Norwegian Correctional Services provided the date of prison release up to 6 months before the date of death. The other social and health services provided data up to 1 year before the date of death. Number of contacts, type of contacts and last contact date were collected from pre-hospital emergency services (ambulance and acute health care clinics), three hospitals that included both psychiatric and somatic wards and community care services in Oslo. Public and private drug treatment facilities, low threshold housing facilities and harm reduction facilities, such as day-time shelters and street clinics, provided contact dates, reason for contact and last contact date. Data were also provided by the public social services in Oslo where all dates for each visit were recorded in addition to complete information on the content of each recorded visit. In the descriptive and logistic regression analysis all contacts in the year prior to death, with any of the included health and social service facilities, were aggregated into one variable that was labelled ‘Contact with any health or social services’ in the year prior to death.

The study was approved by the Norwegian Regional Ethics Committee. Permission was obtained from the Higher Prosecuting Authority to access autopsy reports.

### Data analysis

All analysis was completed using SPSS version 19.0 for Windows. To test for differences between groups, Chi-square analysis was used. In groups with small number Fischer’s exact test was used.

## Results

The majority of cases that died in the study period were men (78%) and the mean age was 37.3 years (SD 10.3) (Table I). Among the cases, 68% were Oslo residents. The most common place of death was a residential address (67%) (Table I). A large proportion (82%) had been in contact with at least one health- and/or social service facility in the year before death. Social services were the service most cases had last been in contact with before death (32%). Median time between last contact and death was 10 days. Mean number of different services the cases had been in contact with in the year before they died was three (range 1–9).

**Table I.** Demographic characteristics, treatment utilization patterns and toxicological findings in those who died from drug-induced overdose in Oslo in the study period.

	Women	Men	Total
	*N* = 51	*N* = 180	*N* = 231
	(100%)	(100%)	(100%)
Mean age (SD)	40.2 (11.5)	36.5 (9.8)	37.3 (10.3)
Oslo residents	42 (82%)	116 (64%)	158 (68%)
Place of death			
Residential address^a^	38 (75%)	117 (65%)	155 (67%)
Outdoors^b^	5 (10%)	36 (20%)	41 (18%)
Institutions^c^	4 (8%)	8 (4%)	12 (5%)
Public buildings^d^	1 (2%)	14 (8%)	15 (7%)
Unknown	3 (6%)	5 (3%)	8 (4%)
Contact with any health- or social services in the year prior to death	47 (92%)	143 (80%)	190 (82%)
Mean number of contacted services (SD)	3.2 (1.8)	3.1 (1.9)	3.1 (1.9)
Released from prison within six months before death	2 (4%)	16 (9%)	18 (8%)
Main intoxicant			
Heroin	27 (53%)	125 (70%)	152 (66%)
Methadone	5 (10%)	19 (11%)	24 (10%)
Buprenorphine	0	1 (1%)	1 (<1%)
Other opioids^e^	14 (27%)	18 (10%)	32 (14%)
Central stimulants	1 (1%)	7 (4%)	8 (4%)
Not poisoning^f^	1 (2%)	5 (3%)	6 (3%)
No toxicology report	3 (6%)	5 (3%)	8 (4%)
Mean number of substances (SD)	3.3 (1.2)	3.1 (1.3)	3.1 (1.3)

a Including shelters.

b Including parking houses and public toilets.

c Including prisons, hospitals, drug treatment facilities.

d Including hotels.

e Includes dextropropokyfen, etylmorphine, fentanyl, codeine, morphine, oxycodon, tramadol.

f Reported as drug-induced death in the National Cause of Death Registry but not in the toxicology reports or hospitals records.

Almost all cases (97%) had undergone post-mortem examination and 94% were examined at the Institute of Forensic Medicine at the University of Oslo, while 3% were examined at a hospital. Heroin was implicated in 155 deaths (67%), and it was considered by the pathologist to be the main intoxicant in 152 cases (66%) (Table I). Of the 38 cases (16%) where methadone was involved, it was determined to be the main intoxicant in 24 cases (10%); seven were in OMT at the time of death (in total, 21 cases were enrolled in OMT at the time of death). Other opioids were found in 39 cases (17%) and in 32 cases (14%) they were determined to be the main intoxicant. According to the post-mortem toxicology reports, polydrug intoxications were common and a single drug was detected in only 10% of the cases. Mean number of substances was 3.1 (range 1–8). Benzodiazepines and/or hypnotics were detected in 69% of the cases and the most common combination of substances was heroin and benzodiazepines and/or hypnotics (50%) (Table II). Of the 38 cases that had used methadone, 32 had combined this with benzodiazepines and/or hypnotics. Ethanol was less common and only 12% had combined heroin and ethanol, and 3% had combined methadone and ethanol.

**Table II.** Combinations of substances detected in blood samples^a^.

	Heroin	Methadone	Buprenorphine	Stimulants^b^	Cannabis	Ethanol	Other opioids^c^	Paracetamol	Benzodiazepines and/or hypnotics^d^	Psychiatric prescription drugs^e^
Heroin	**155**									
Methadone	12	**38**								
Buprenorphine	3	0	**4**							
Stimulants^b^	61	15	**3**	**78**						
Cannabis	32	11	1	16	**41**					
Ethanol	27	8	0	14	7	**45**				
Other opioids^c^	3	6	0	6	3	13	**39**			
Paracetamol	0	0	0	0	1	6	18	**18**		
Benzodiazepines and/or hypnotics^d^	115	32	4	60	36	27	83	14	**160**	
Psychiatric prescription drugs^e^	34	10	0	18	5	8	29	6	42	**54**

a The table shows the number of cases reported to have had the combination of substances detected in blood and not what was determined to be main intoxicant. The median number of drugs detected in the blood samples in each case was 3, thus the number in this table will sum up to more than 231.

b Includes cocaine, amphetamine, methamphetamine, ecstasy.

c Includes fentanyl, karisoprodol/meprobamat, ketobemidon, oxycodon, kodein, dekstropropoksyfen, morphine.

d Includes flunitrazepam, diazepam, nitrazepam, alprazolam, oxazepam, klonazepam, fenazepam, alimemazin, prometazin, zolpidem, zopiclone.

e Includes amitriptylin/nortriptilyn, paroxetin, trimipramin, citalopram, venlafaxin, mirtazapin, fluoxetin, sertralin, mianserin, doxepin, duloksetin, lamotrigin, karbamazepin, olanzapin, levomepromazin, klorprotixen, hydroxyzin, amisulprid, zuclopentixol.

One distinct subpopulation among the cases was the 51 women. The women were on average almost 4 years older (40.2 vs. 36.5 years) when they died. In addition, there were more women who were Oslo residents (82% vs. 64%, X^2^ = 5.9, *p* = 0.015). Furthermore, a significantly higher proportion of women had been in contact with health- or social services in the year prior to death (92% vs. 80%, X^2^ = 4.4, *p* = 0.036). Although heroin was the most common main intoxicant considered by the pathologist in both women and men, fewer women died from heroin (53% vs. 70%, X^2^ = 4.8, *p* = 0.028). Instead, more women died from other opioids such as morphine, oxycodon and tramadol (27% vs. 10%, X^2^ = 10.1, *p* = 0.001).

Another distinct subpopulation were those that were registered in the Directory of Residence with a home address outside of the capital (non-Oslo residents) (*n* = 73). There were a higher proportion of men in this subpopulation (36% vs. 18%, X^2^ = 5.9, *p* = 0.015). They were slightly younger (32.7 vs. 39.4 years) and heroin was the main intoxicant in a higher proportion of this population (86% vs. 56%, X^2^ = 19.9, *p* < 0.001). A higher proportion were recently released from prison (16% vs. 8%, X^2^ = 11.1, *p* = 0.001). Furthermore, fewer non-Oslo residents had been in contact with any health and social services in the year prior to death (66% vs. 90%, X^2^ = 19.9, *p* < 0.001).

Additionally, the small proportion (*n* = 18) that had been released from prison within 6 months prior to death made up a subpopulation. The median time from prison release to death was 18 days (range 0–182). Ten cases died within 3 weeks after release; eight of these died within the first 2 weeks. There were only two women among the 18 cases. Mean age was 32.1 years (SD 7.3), and only 33% were Oslo residents. Half of the cases were found outdoors or in a public building. Only one of the cases had not been in contact with any health and social services in the year before death, and the median time between last contact and death was 3.5 days. In this subpopulation, emergency care services were the most common service they last contacted before they died (47%), whereas in only three cases (18%), social services were the last contact facility. Heroin intoxications were according to the pathologist the most common main cause of death (83%). In the three cases where heroin was not the main intoxicant, one died from morphine intoxications and in two cases there were no toxicological information available.

Furthermore, the 34 cases that died within a year after discharge from inpatient drug treatment such as detoxification and long-term residential rehabilitation made up a fourth subpopulation. The median time from discharge to death was 102 days. Ten cases died within 3 weeks after discharge; of these six died in the first 2 weeks. The mean age was 35.3 years (SD 11.0) and 77% were Oslo residents. Among these cases, 31% were found outdoors or in a public building. As in the other described subpopulations, the most common main intoxicant considered by the pathologist was heroin (71%). In addition, eight cases died in the year after discharge from OMT, the first death occurred after 48 days and the median time from discharge to death was 108 days.

## Discussion

Men in their mid- to late 30s made up the majority of cases and a residential address was the most common place of death. Heroin was implicated in 67% of the cases and the majority were polydrug intoxications. More than half of the cases had contact with health- and/or social services often in close proximity of the death. Women were on average four years older, more often Oslo residents and fewer died from heroin intoxication than men. Non-Oslo residents were younger and more likely to have been found outdoors with heroin as the main intoxicant. Other identified subpopulations were those who died after prison release and those discharged from drug treatment.

The majority died from a heroin overdose and most overdoses were polydrug intoxications. In a study among IDUs in Oslo, almost all reported combined use with benzodiazepines, whereas less than half reported combined use with alcohol [22]. The high proportion of heroin combined with benzodiazepines and the relatively few cases where ethanol was implicated are therefore likely to mirror the patterns of drug use among drug users in Norway. The majority of heroin users in Norway inject [23], and Norway has a higher proportion of IDUs compared to many other European countries [15]. In addition, Norway has a high proportion of polydrug users [22]. The high prevalence of injecting heroin use and polydrug use, especially benzodiazepine use, are likely to be factors which in combination contribute to the high annual number of drug-induced deaths.

The finding that a majority of cases (82%) had been in contact with health and social services is in accordance with a similar study from Glasgow in 1999 [24]. Jones and colleagues found that as many as 90% of their cases had visited a general practitioner in the year before they died, and 60% more than six times [24]. These findings suggest that many of those who die are available for preventive measures through their contact with health and social services. However, these findings show that contact with such services not necessarily prevent overdoses, instead specific preventive measures need to be introduced. These services are therefore important for establishing contact and introducing preventive measures.

The proportion of women was similar to the proportion of female injecting drug users in Norway [25], but they differed significantly from men in regards to age, capital residency and toxicology. Female injectors are known to have a slightly different drug using pattern than men [26–28], and the findings in toxicology are therefore likely to mirror this. Although women make up only a small proportion of those who die from overdoses, it is still important that they are addressed specifically when preventive measures are designed and implemented, in order to reach their specific needs.

A minority of drug-induced deaths are street-events [3,8,29], there exists, however, extensive evidence of an increased mortality risk among those who are homeless or shelter users [11,12,30]. In this study, non-Oslo residents were younger, more likely to have been found outdoors with heroin as the main intoxicant, to not have had contact with health and social services and to have been recently released from prison. It is therefore possible that some of the non-Oslo residents were homeless or shelter users and thereby had an increased mortality risk. A person who is a non-resident may not have the same access to services in the city as residents. Thus, this population should be targeted specifically and potentially in a different manner than homeless and shelter users who are city residents.

Other identified subpopulations were those who died after prison release and those discharged from drug treatment. The various subpopulations found among the cases show that those who die are a heterogenic population. The heterogeneity makes it difficult to implement a single set of preventive measures as there is a whole range of factors that alone or in combination will need to be addressed. Women will need to be addressed differently than men, and non-Oslo residents need to be managed differently than city residents. Those who are released from prison or discharged from drug treatment need to be addressed specifically. In addition, the high prevalence of heroin intoxications in combination with benzodiazepines should also be addressed specifically. Importantly, the combination of contributing factors is likely to increase the possibility of death. It is therefore important to identify and address those who are exposed to combinations of contributing factors. The high proportion of cases that had been in contact with health and social services suggests that it is possible to identify those at particular risk within these services and to reach a large proportion of the population at risk with preventive measures if such measures are introduced. Although preventive interventions need to be multifaceted and address the heterogeneity of those who die from overdose, they also need to be implemented in conjunction with one another.

### Strengths and limitations

The findings from this study have certain limitations. In this observational study of cases we were not able to estimate risk. Additionally, the number of contacts assessed in this study is likely to be underestimated as not all services, such as ambulance services and various social services, always record all contacts. Some of the findings, such as the gender distribution, the different places of death and the proportion who were out of treatment, released from prison or discharged from treatment were similar to other studies with a similar design [3,8,24]. However, it is important to be aware that the proportion of IDUs and heroin users may vary from setting to setting, and comparisons between settings should therefore always be made with caution. The main strength of this study is the inclusion of all deaths diagnosed as drug-induced deaths in an urban area. Importantly, the high proportion of persons who underwent a post-mortem examination supports the high reliability of the information on the underlying causes of death. Furthermore, the large network of health and social services that provided information ensured that it was possible to detect possible patterns and subgroups among the deceased.

## Conclusion

The findings presented suggest that the majority of those who died would have been available for preventive measures through their contacts with health and social services. This highlights the importance these services could play in implementing preventive measures in the future. Most cases were exposed to a range of contributing factors which suggests that health and social services need to identify and address those individuals who are at particular risk; especially those with multiple risk factors present at the same time. Although the findings highlighted the heterogeneity of those who died and preventive measures therefore need to be multifaceted, it is important that preventive measures are implemented in conjunction with one another. Finally, it is important for policymakers and health and social workers in various countries to consider subpopulations such as women and non-city residents when developing public health interventions to prevent overdose deaths.
